# General Anesthetics in CAncer REsection Surgery (GA-CARES) randomized multicenter trial of propofol vs volatile inhalational anesthesia: protocol description

**DOI:** 10.1186/s13741-022-00290-z

**Published:** 2023-01-11

**Authors:** Elliott Bennett-Guerrero, Jamie L. Romeiser, Samuel DeMaria, Jacob W. Nadler, Timothy D. Quinn, Sanjeev K. Ponnappan, Jie Yang, Aaron R. Sasson

**Affiliations:** 1 Department of Anesthesiology, Renaissance School of Medicine at Stony, Brook University, Stony Brook, NY USA; 2 Department of Anesthesiology, Perioperative and Pain Medicine, The Icahn School of Medicine at Mount Sinai, New York, NY USA; 3 Department of Anesthesiology and Perioperative Medicine, University of Rochester School of Medicine, New York, NY USA; 4 Department of Anesthesiology, Preoperative Medicine and Pain Medicine, Roswell Park Comprehensive Cancer Center, Buffalo, NY USA; 5 Department of Anesthesiology, Long Island Jewish Medical Center at Northwell Health, New Hyde Park, NY USA; 6 Department of Family, Population and Preventive Medicine, Renaissance School of Medicine at Stony Brook University, Stony Brook, NY USA; 7 Department of Surgery/Surgical Oncology, Renaissance School of Medicine at Stony, Brook University, Stony Brook, NY USA

**Keywords:** Cancer surgery, General anesthesia, Propofol, Volatile anesthesia

## Abstract

**Background:**

Studies indicate that patients can be “seeded” with their own cancer cells during oncologic surgery and that the immune response to these circulating cancer cells might influence the risk of cancer recurrence. Preliminary data from animal studies and some retrospective analyses suggest that anesthetic technique might affect the immune response during surgery and hence the risk of cancer recurrence. In 2015, experts called for prospective scientific inquiry into whether anesthetic technique used in cancer resection surgeries affects cancer-related outcomes such as recurrence and mortality. Therefore, we designed a pragmatic phase 3 multicenter randomized controlled trial (RCT) called General Anesthetics in Cancer Resection (GA-CARES).

**Methods:**

After clinical trial registration and institutional review board approval, patients providing written informed consent were enrolled at five sites in New York (NY) State. Eligible patients were adults with known or suspected cancer undergoing one of eight oncologic surgeries having a high risk of cancer recurrence. Exclusion criteria included known or suspected history of malignant hyperthermia or hypersensitivity to either propofol or volatile anesthetic agents. Patients were randomized (1:1) stratified by center and surgery type using REDCap to receive either propofol or volatile agent for maintenance of general anesthesia (GA). This pragmatic trial, which seeks to assess the potential impact of anesthetic type in “real world practice”, did not standardize any aspect of patient care. However, potential confounders, e.g., use of neuroaxial anesthesia, were recorded to confirm the balance between study arms. Assuming a 5% absolute difference in 2-year overall survival rates (85% vs 90%) between study arms (primary endpoint, minimum 2-year follow-up), power using a two-sided log-rank test with type I error of 0.05 (no planned interim analyses) was calculated to be 97.4% based on a target enrollment of 1800 subjects. Data sources include the National Death Index (gold standard for vital status in the USA), NY Cancer Registry, and electronic harvesting of data from electronic medical records (EMR), with minimal manual data abstraction/data entry.

**Discussion:**

Enrollment has been completed (*n* = 1804) and the study is in the follow-up phase. This unfunded, pragmatic trial, uses a novel approach for data collection focusing on electronic sources.

**Trial registration:**

Registered (NCT03034096) on January 27, 2017, prior to consent of the first patient on January 31, 2017.

## Background

Cancer recurrence occurs in approximately one-third of patients who have undergone a cancer resection surgery. Previous studies indicate that patients can be “seeded” with their own cancer cells during surgery, and that the immune response to this “seeding” might influence the risk of cancer recurrence (Eschwege et al. 1995; Bij et al. 2009; Yamaguchi et al. 2000; Tohme et al. 2017; Fodale et al. 2014; Kim 2017; Sekandarzad et al. 2017; Stollings et al. 2016; Tedore 2015). In this setting, a pro-inflammatory milieu may be beneficial since the activated immune system can combat circulating cancer cells more effectively (Tohme et al. 2017; Fodale et al. 2014; Kim 2017; Sekandarzad et al. 2017; Stollings et al. 2016; Tedore 2015). Consistent with this theory, immunosuppression, e.g., from blood transfusion, has been shown in some analyses to be associated with worse outcomes after cancer surgery across multiple cancer types (Abe et al. 2017; Boshier et al. 2018; Lopez-Aguiar et al. 2019; Luan et al. 2014; Nakanishi et al. 2019; Wang et al. 2015; Xun et al. 2018).

Growing evidence suggests that the anesthetic technique used during surgery might affect the immune response during surgery (Tohme et al. 2017; Fodale et al. 2014; Kim 2017; Sekandarzad et al. 2017; Stollings et al. 2016; Tedore 2015). Volatile agents, for example, may impair or suppress the function of natural killer cells and upregulate HIF-1, both of which may promote tumor metastasis. In contrast, propofol may preserve natural killer-cell function and downregulate HIF-1. Retrospective data, however, provide inconclusive results as to whether propofol provides an advantage in long-term survival over volatile agents (Jin et al. 2019; Yap et al. 2019). Due to the lack of prospective randomized data comparing propofol vs gas in cancer resections, there is no conclusive evidence to support one practice or the other.

In 2015, experts in the field (*British Journal of Anesthesiology Workgroup on Cancer and Anesthesia* and the *American Society of Anesthesiologists*) (Buggy et al. 2015) called for prospective scientific inquiry into whether anesthetic technique used in cancer resection surgeries plays a role in cancer-related outcomes such as recurrence and mortality. In response to this call, in 2017, we began a large Phase 3 multicenter randomized control study called the *G*eneral *A*nesthetics in *Ca*ncer *Res*ection (*GA-CARES*) trial. To date, our study is one of only four large-scale outcome trials across the globe in this field: NCT02660411 (*n* = 1228 actual), NCT01975064 (*n* = 8000 projected), NCT04316013 (*n* = 5736 projected).

## Methods/design

### Objectives/hypothesis

The primary objective of this study is to test the hypothesis that the administration of propofol for maintenance of general anesthesia during cancer surgery improves overall survival (minimum 2-year follow-up) compared with general anesthesia maintained with a volatile agent. Secondary objectives include cancer recurrence and postoperative hospital length of stay.

### Trial design

Our goal is to conduct a pragmatic trial (Patsopoulos 2011), which is also sometimes referred to as a large simple trial. In contrast to explanatory trials, which attempt to minimize variability and optimize efficacy in a more ideal setting than the actual clinical setting, pragmatic trials ask the question “What does this intervention do in the real world?” A pragmatic, large, multicenter trial desires to have high external validity/generalizability. Pragmatic trials, therefore, have few exclusion criteria and promote patients receiving routine care, with the exception of the study intervention to which they are randomized. In addition, there are usually few extraneous study procedures, e.g., blood draws.

In GA-CARES, there were no changes to the routine care of enrolled patients other than randomization to propofol vs. volatile agent for the maintenance of general anesthesia, which all patients required as part of their routine care.

The trial is a multicenter, parallel arm, partially blinded, randomized trial.

### Ethics, registration, and safety monitoring (DSMB)

The trial was registered (NCT03034096) on January 27, 2017, prior to consent of the first patient on January 31, 2017.

Ethics/Institutional Review Board (IRB) approval was obtained from the independent IRB at each of the five study sites in New York State: (1) Renaissance School of Medicine at Stony Brook University (lead site and coordinating center), (2) Icahn School of Medicine at Mount Sinai, (3) University of Rochester, (4) Roswell Park Comprehensive Cancer Center, (5) Long Island Jewish Medical Center at Northwell Health. These 5 independent IRBs agreed that there is an equipoise to randomize patients to these two commonly used anesthetic techniques. For example, there are conflicting results from observational analyses, national organizations have called for RCTs on this topic, and there are three other large multicenter trials (some government-funded) studying the same intervention.

The trial has an independent Data and Safety Monitoring Board (DSMB) comprised of three individuals not otherwise involved in the trial nor from the institutions where the trial is being conducted. This DSMB met every 6 months to review trial data, and after each meeting recommended continuation of the trial without any modifications.

### Eligibility criteria

*Inclusion criteria:* Adult patients undergoing a diverse group of surgical oncologic procedures under general anesthesia were eligible. More specifically, patients with known or suspected cancer and scheduled to undergo any of the following procedures were eligible: (1) Lobectomy or pneumonectomy, (2) esophagectomy, (3) radical (total) cystectomy; (4) pancreatectomy, (5) partial hepatectomy, (6) gastrectomy (subtotal or total), (7) cholecystectomy or bile duct resection for known or suspected cancer, (8) hyperthermic intraperitoneal chemotherapy (HIPEC). These procedures were chosen because they represent biologically aggressive cancers and have relatively poor cancer-related outcomes.

Exclusion criteria were as follows: (1) age less than 18 years; (2)American Society of Anesthesiology (ASA) Class 5; (3) projected life expectancy less than 30 days; (4) known or suspected hypersensitivity to either propofol, e.g., egg or soy allergy, or volatile general anesthetic agents; (5) Known or suspected history of malignant hyperthermia; (6) previously randomized in the GA-CARES trial; (7) carcinoid, neuroendocrine, and gastrointestinal stromal tumors (GIST).

### Study intervention

After written informed consent was obtained, patients were randomized (1:1) using a REDCap randomization module to propofol or a volatile inhalational anesthetic for maintenance of general anesthesia. The specific volatile agent used (isoflurane, sevoflurane, or desflurane) was determined by the clinician. Observational data do not indicate differences between these agents in cancer outcomes. The type of volatile agent(s) used and maximum volatile gas concentration were collected for potential exploratory analyses. It was not feasible to collect the “area under the curve” volatile exposure for each subject. Total intraoperative propofol dose was collected in all subjects. Propofol (bolus dosing) was allowed for induction of general anesthesia in both study arms. Centers that routinely use bispectral index (BIS) were instructed to titrate these anesthetics to a BIS of 40–60 consistent with previous studies supporting its use (Avidan et al. 2008).

### Concomitant medications/procedures

Since this is a pragmatic trial, there were no standardization/protocol requirements regarding the use of opioids, regional anesthesia, or other concomitant medications/procedures. There were no blood draws. Surgical management was not standardized by the trial in any way. These and other potential confounders, however, were recorded and analyzed to confirm the anticipated balance in these variables between study arms given the large sample size.

### Blinding

It was not possible to blind the anesthesia care team to study arm in this trial. The surgical team could obtain this information, but it is unlikely this would affect their surgical management of the patient. The trial’s primary outcome is all-cause mortality, which is an objective measure and unlikely to be affected from lack of blinding. For the trial’s secondary endpoint of cancer recurrence, NY State Cancer Registry staff at each site are functionally blinded, since abstractors do not know whether a patient is in the trial, and even if they did, it is unlikely this would affect their data abstraction.

### Data management

As described below, the main sources of data are (1) Hospital EMR, (2) NY Cancer Registry, and (3) National Death Index:

1. *Electronic medical record*: Much of the data related to the surgery and hospitalization is harvested from the intraoperative/anesthesia and hospital electronic medical record (EMR). Obtaining these data electronically/directly from the EMR minimizes potential transcription errors given that the EMR is already the source document for “source document verification” of most variables. Electronic harvesting of these data also minimizes effort by the site’s research staff since manual data abstraction and data entry are time-consuming. To achieve this, the local site obtains electronic reports for enrolled subjects in excel or csv format, and these are transferred securely via an encrypted BOX folder to the coordinating center. These data are housed on a Stony Brook Medicine Information Technology (SBMIT) server that can only be accessed by study personnel and is backed up on a regular basis per SBMIT protocols. Extracts that have been sent to the coordinating center are matched, merged, and imported into each site’s REDCap database, as well as for verification of any possible protocol deviations. Two of the sites (LIJ/Northwell and Roswell) were not able to provide the requested data electronically and thus entered these data manually into their site-specific REDCap database. To prevent each site from being able to access data from other sites, the REDCap database for the trial was cloned into site-specific databases, each with identical data elements, so each study site had access to only their own patients’ data. Data definitions for EMR elements and manually entered data elements were consistent across each site.
2. *New York Cancer Registry*: By only including centers in NY State, we have the ability to leverage the NY Cancer Registry for collection of important variables. Per NY State Law, the NY Cancer Registry must collect, process, and report information about all New Yorkers diagnosed/treated with cancer. Data variables for collection include sociodemographic characteristics, disease/tumor-related characteristics (e.g., histological information such as staging, lymph node involvement), and treatment information (e.g., dates of surgical treatment, chemotherapy, and radiation treatments). Information at all NY hospitals is collected and coded using strict procedures and consolidated for routine reports at the state level. The NY Cancer Registry follows patients annually for life to assess for recurrence and mortality.
  The quality, completeness, and timeliness of the registry data are submitted and verified annually by the North American Association of Central Cancer Registries (NAACCR) certification process. The NY State Cancer Registry, which has been collecting information on patients with cancer for more than 50 years, has consistently received a Gold-level certification for each year of data from 1998 through the present. Each of the five centers in the GA-CARES trial has a specific NYS Cancer Registry contact who is able to supply extracts for the site’s enrolled patients. By leveraging the use of the cancer registry, a resource that is partially funded by the CDC and NCI, our trial can ensure the consistency of definitions and coding procedures across these data variables at all five sites. This provides an extra layer of quality control that is critical for the success of a multicenter trial.
  Under the direction of one of the trial’s data managers, NY Cancer Registry data extractions are coordinated with each study site’s affiliated cancer registry office. Labor-intensive matching and selection processes for GA-CARES study participants’ records are performed and merged into each site’s REDCap database.
3. *National Death Index (NDI)*: The NDI will be queried for survival data. The NDI is a centralized database of death record information on file in state vital statistics offices. Working with these state offices, the National Center for Health Statistics (NCHS) established the NDI as a resource to aid epidemiologists and other health and medical investigators with their mortality ascertainment activities (https://www.cdc.gov/nchs/ndi/index.htm). Of the available national mortality databases, the NDI has been demonstrated to have the highest sensitivity for recording mortality (Cowper et al. 2002). Access to NDI records is relatively inexpensive, i.e., 21 cents per subject per year, for date of death and cause of death.

In addition to the above sources of data, study staff manually record a small number of variables not easily obtained by the EMR and NY Cancer Registry. For example, postoperative ICU admission can be reliably assessed through a review of notes in the EMR; however, this variable may not be easy to harvest electronically at some sites if the hospital does not have a standardized data field for “ICU admission- yes vs no”.

### Statistical methods

*Data collection/management*, employing REDCap, has been described above.

### Randomization and allocation concealment

Block randomization with random permuted block sizes of 4, 6, 8, 10, and 12 was used to ensure a similar number of subjects in each arm over time while minimizing predictability. Randomization was first stratified by site, then stratified by the 8 procedure types listed in the inclusion criteria. All randomization lists were uploaded to each site’s REDCap database by the trial’s data manager. Randomization was performed as soon as possible to surgery using REDCap’s secure interactive web-based randomization system (IWRS), which provided for excellent allocation concealment. Since we randomized patients very close to surgery, it is not surprising that we observed very few “randomization failures”, i.e., over 98% of consented patients were randomized, and very few (approximately 1.2%) randomized patients had surgery canceled or withdrew from the study.

### Study endpoints

The primary and key secondary endpoints are consistent with consensus definitions for standardized endpoints for surgical cancer outcome trials (Buggy et al. 2018). The *primary endpoint* is *all-cause mortality* with a follow-up of at least 2 years (maximum 5 years) after surgery. All-cause mortality is a clinically significant and objective endpoint that is well accepted for cancer trials such as this (Buggy et al. 2018). Mortality can be ascertained reliably through various sources including querying the National Death Index registry, which is the gold standard for vital status in the USA (as described above). The other three similar ongoing multicenter RCTs also use overall survival as either the primary endpoint (NCT02660411, NCT01975064) or as a key secondary endpoint (NCT04316013).

Another advantage of using mortality as the primary endpoint is that it is still relevant even if patients are never disease free. It is possible that use of one anesthetic drug, e.g., propofol, might reduce circulating cancer cells and thus overall burden of disease, leading to longer survival, even in patients who are never disease free. Indeed, in more advanced disease, there are probably more circulating tumor cells and the impact of minimizing immunosuppression might even be greater.

Our key secondary endpoint is cancer recurrence. This endpoint is important but is more challenging to ascertain, especially if patients move to another state or are lost to follow-up. This is less of a concern with an all-cause mortality endpoint, which can be reliably obtained from a national registry (NDI) (Cowper et al. 2002). Moreover, some elective surgical patients are never “cancer free”, i.e., they have surgery with curative intent but unresectable disease is observed. These patients cannot be assessed for recurrence as they were never disease free.

Postoperative length of hospital stay will also be recorded and analyzed. It is a reasonable surrogate for immediate postoperative outcome, which we believe will be similar between study groups based on Pasin et al.’s meta-analysis of short-term mortality after propofol vs. volatile anesthesia (95 studies, *n* = 9806 total patients) (Pasin et al. 2015).

### Sample size/power

Because death information can be accurately assessed using the National Death Index (Cowper et al. 2002), all randomized patients will be included in the intention-to-treat (ITT) analysis for the primary objective. There are limited national data on mortality after elective cancer surgery for all eight types of surgical procedures eligible for the study. National cancer statistics (2009–2015), which include all patients (not just elective surgery), show 5-year survival rates (lowest to highest) of 9% (pancreas), 13% (esophagus), 15% (liver), 17% (lung), 32% (stomach), and 65% (bladder). These lower survival rates, however, are for 5 years, not 2 years of follow-up, which was the basis for our sample size calculation. They also include many patients with widespread unresectable disease at presentation who are not eligible for surgery. Moreover, advances in cancer care, e.g., immunotherapy, are improving many of these dismal prognoses. Therefore, we assumed that we would observe a blended mortality rate of at least 15% at 2 years (i.e., observed survival 85%) in the volatile arm and 10% mortality (observed survival of 90%) in the propofol arm, with a 5% absolute difference being a small but clinically significant difference in outcome given the ease and low cost of using propofol for these cases if propofol is shown to be better.

The estimated study power using two-sided log-rank tests at a significance level of 0.05 according to different assumed 2-year overall survival rates at each arm is listed in Table 1. The power estimation was carried out using PASS 12 (Kaysville, Utah). Stratification factors were not used in the design phase because of the absence of a priori knowledge about the design parameters like effect size within each stratum. If the baseline survival rate at 2 years post-surgery used for unstratified design is close to the weighted average of all stratum-specific rates with the patient allocation proportions as weights, then type I error is maintained and the power loss is negligible (Srivastava et al. 2007).

**Table 1:**
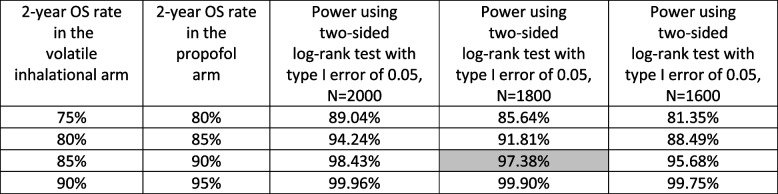
Power and sample size

The table shows power to detect different overall survival (OS) using 1600, 1800, and 2000 patients with different assumptions for 2-year overall survival rate. A clinically relevant absolute difference in 2-year overall survival is considered to be 5%. No interim analyses are planned. Base case assumed for this trial is highlighted in gray (97.38%)

### Statistical analyses

All statistical analyses will be performed by a statistics analytic team using SAS 9.4 (SAS Institute Inc., Cary, NC). The primary objective (all-cause mortality) will be analyzed using an *ITT analysis*, i.e., all patients randomized will be included in this primary analysis. Time to overall survival is defined as from the date of randomization to the death time or the last follow-up date, whichever occurs first. Kaplan–Meier curves of overall survival will be constructed for patients in each arm. Exact stratified log-rank test will be applied for the primary objective of comparing overall survival using two stratification factors (study site and procedure type) from the randomization step. As an exploratory analysis, Cox’s proportional hazard model will be used to make the comparison in overall survival after adjusting for study site, procedure type, and other possible confounding factors. The sample size allows for possible subgroup analyses including within each cancer type, cancer stage, age groups, and gender. Cancer recurrence will be analyzed similarly with time to recurrence defined as from the date of randomization to the death time, the date of disease recurrence, or the last follow-up date, whichever occurs first. Cancer recurrence can only be assessed in patients who had cancer at the time of surgery and underwent a curative resection where they were deemed cancer free at that time. Postoperative hospital length of stay (LOS) will be compared using a stratified *t*-test and explored using a multiple linear regression model to adjust for possible confounding factors including study site and procedure type. Depending on the model fitting diagnosis, a generalized regression model assuming LOS follows a negative binomial distribution may be used instead. Model assumptions will be diagnosed and data transformation may be needed in order to get assumptions met. All analyses will also be performed on a “per-protocol” analysis population defined as patients with a confirmed cancer diagnosis who undergo surgery and receive the correct anesthesia agent as assigned.

## Discussion

GA-CARES (NCT03034096) is the first large multicenter outcome trial in the USA and is one of four large trials (NCT02660411, NCT01975064, NCT04316013) on this important question (Table 2—summary of trials). Two of these four trials have completed enrollment. The four trials are similar in many respects but several important differences warrant comment. All four trials are similar in that they compare propofol with volatile agent for maintenance of general anesthesia during cancer surgery. In addition, most have a relatively pragmatic design that addresses the impact of this intervention in the “real world” setting, which is critical to ensure generalizability of the results. Three of the four trials, including GA-CARES, chose overall survival/all-cause mortality as the primary endpoint, which reflects the relevance of this “hard” endpoint for cancer outcome trials such as these. One of the trials chose disease-free survival as their primary endpoint, but specifies all-cause survival endpoint as their key secondary endpoint.

**Table 2:** Multicenter RCTs comparing impact of propofol vs. volatile anesthesia on survival after cancer surgery

Sponsor country	Study arms	*n* =	Eligible patients	Outcomes P = primary S = secondary	Trial registration	Trial status
China	Sevoflurane vs propofol	1228 (actual)	Surgery for primary malignant tumor; adults 65–90 years; no radiation or chemotherapy preoperatively; no neurologic disorders; no significant organ dysfunction	P: all-cause survival (up to 5 y) S: recurrence free survival (5 y); quality of life (3 y); cognitive function (3 y)	NCT02660411	Enrollment complete
USA	Volatile (sevoflurane, desflurane, isoflurane) vs. propofol	1804 (actual)	Lung, esophageal, pancreatic, radical bladder, liver, gallbladder; adults ≥ 18 years	P: all-cause survival (2–5 y) S: cancer recurrence (2–5 y); postoperative length of stay	NCT03034096	Enrollment complete
Sweden	Sevoflurane vs propofol	8000 (target)	Radical breast or colorectal cancer surgery; adults ≥ 18 years	P: all-cause survival (5 y) S: all-cause survival (1 y)	NCT01975064	Enrolling
Australia	Sevoflurane vs. propofol; factorial design also randomizes to lidocaine infusion or not	5736 (target)	Stage I–III colorectal or stage I–IIIa NSC lung cancer; distant metastases, no significant liver disease; not receiving medications that are CYP1A2 or CYP3A4 inhibitors	P: disease free survival (3 y) S: all-cause survival (3 y); postoperative complications (5 days postoperatively); chronic post surgical pain (90 days and 12 months)	NCT04316013	Enrolling

*RCT* Randomized controlled trial, *y* Years follow

Notwithstanding the above, there are some differences in these trials with regard to the types of cancer surgeries eligible for enrollment. One trial (NCT02660411) does not specify a cancer surgery type, and two of the other trials (NCT01975064, NCT04316013) allow enrollment of patients with primary colorectal surgery. GA-CARES, in contrast, focuses on surgeries with biologically aggressive cancers and poor cancer outcomes, e.g., the pancreas, esophagus, stomach, and lung, which is anticipated to result in higher event rates. GA-CARES investigators chose to not enroll patients undergoing breast or colorectal surgery since those eligible for surgery have generally good outcomes, compared with individuals with those cancers who present with extensive disease and are not surgical candidates. Having a greater number of events (i.e., deaths) reduces the risk that the trial is negative due solely to insufficient power. Moreover, since it has been suggested that a differential effect of anesthetic technique is more likely to be manifested in larger more morbid cancer surgeries (Sessler and Riedel 2019). In an editorial, Sessler and Riedel wrote “Although tumor type may play a role, available data seem most consistent with the theory that the magnitude of surgical stress is a key driver.” and “Available data thus suggest that to the extent that propofol–total intravenous anesthesia reduces cancer recurrence and improves survival, benefit is most probable in patients having major cancer surgery” (Sessler and Riedel 2019). This potentially relevant issue was postulated by Sessler et al. to explain the negative result from their RCT of GA vs regional (paravertebral block) anesthesia for breast surgery where surgeries were often minor and postoperative recurrence rates/mortality rates were relatively low (Sessler et al. 2019).

It is important to note that another advantage of using mortality as the primary endpoint is that it is still relevant if patients are never disease free, e.g., their surgery was unable to be curative. In other words, it is possible that use of one anesthetic drug, e.g., propofol, might reduce circulating cancer cells and thus overall burden of disease, leading to longer survival, even in patients who are never disease free. Indeed, in more advanced disease, there are probably more circulating tumor cells and the impact of minimizing immunosuppression with propofol may even be greater. This was one of our justifications for allowing patients with metastatic disease to participate in GA-CARES, as we felt they might also benefit from a certain type of anesthesia. Another justification for also including patients with metastatic disease in our trial was observational data from Wigmore et al. where the type of general anesthesia (propofol vs volatile inhalational) was associated with 1-year mortality in patients with and without metastatic disease (Wigmore et al. 2016).

It is important to emphasize that GA-CARES, and to a large extent the other three trials (Table 2), are *pragmatic trials* (Patsopoulos 2011; Ford and Norrie 2016). In contrast to *explanatory* or *exploratory* trials, which attempt to minimize variability and optimize efficacy in an often artificial ideal setting, pragmatic trials focus on the “real world” clinical setting with “real world patients”. Pragmatic trials have few exclusion criteria and promote patients receiving routine care with the exception of the study intervention to which they are randomized. As described in a review: “Pragmatic trials are designed to evaluate the effectiveness of interventions in real-life routine practice conditions, whereas explanatory trials aim to test whether an intervention works under optimal situations. Pragmatic trials produce results that can be generalized and applied in routine practice settings. Since most results from exploratory trials fail to be broadly generalizable, the “pragmatic design” has gained momentum” (Patsopoulos 2011). Therefore, pragmatic trials strive for high external validity, which increases the ability to generalize the study results to a broader population.

In addition, pragmatic trials also have additional benefits including lower cost/resource utilization and more rapid enrollment. It is important to stress that pragmatic trials are not inherently better or worse than explanatory trials; they merely ask and answer different study questions. For the question of whether it matters if one uses propofol vs. volatile anesthesia in cancer surgery, we, and the other ongoing trials, believe the pragmatic approach is more relevant to clinicians and those who draft practice guidelines.

GA-CARES has certain design elements that are novel. There has been much discussion of how electronic medical records can in theory be leveraged for data collection in prospective clinical outcome trials (Mc Cord and Hemkens 2019). Most clinical trials, however, still use the traditional model of manual data collection into study-specific data collection tools. We chose to collect most of our data from high-quality electronic sources, e.g., EMR, NY State Cancer Registry, and NDI. We believe that this will increase the quality and completeness of data, while also being less resource intensive. It is important to note, that while this does appear to decrease effort at the local site level, it shifts some of this effort to the coordinating center, where there are greater demands for importing, merging, and checking the data extracts.

## Data Availability

Not applicable (Methods publication only).
